# Renal sympathetic denervation improves pressure-natriuresis relationship in cardiorenal syndrome: insight from studies with Ren-2 transgenic hypertensive rats with volume overload induced using aorto-caval fistula

**DOI:** 10.1038/s41440-024-01583-0

**Published:** 2024-02-02

**Authors:** Zuzana Honetschlägerová, Zuzana Husková, Soňa Kikerlová, Janusz Sadowski, Elzbieta Kompanowska-Jezierska, Miloš Táborský, Zdenka Vaňourková, Petr Kujal, Luděk Červenka

**Affiliations:** 1 Center for Experimental Medicine, Institute for Clinical and Experimental Medicine, Prague, Czech Republic; 2 Department of Renal and Body Fluid Physiology, Mossakowski Medical Research Institute, Polish Academy of Sciences, Warsaw, Poland; 3 Department of Internal Medicine I, Cardiology, University Hospital Olomouc and Palacký University, Olomouc, Czech Republic; 4 Department of Pathology, 3rd Faculty of Medicine, Charles University, Prague, Czech Republic

**Keywords:** Volume-overload heart failure, Ren-2 transgenic hypertensive rat, Renal autoregulation, Renal blood flow, sodium excretion

## Abstract

The aim was to evaluate the effects of renal denervation (RDN) on autoregulation of renal hemodynamics and the pressure-natriuresis relationship in Ren-2 transgenic rats (TGR) with aorto-caval fistula (ACF)-induced heart failure (HF). RDN was performed one week after creation of ACF or sham-operation. Animals were prepared for evaluation of autoregulatory capacity of renal blood flow (RBF) and glomerular filtration rate (GFR), and of the pressure-natriuresis characteristics after stepwise changes in renal arterial pressure (RAP) induced by aortic clamping. Their basal values of blood pressure and renal function were significantly lower than with innervated sham-operated TGR (*p* < 0.05 in all cases): mean arterial pressure (MAP) (115 ± 2 vs. 160 ± 3 mmHg), RBF (6.91 ± 0.33 vs. 10.87 ± 0.38 ml.min^–1^.g^–1^), urine flow (UF) (11.3 ± 1.79 vs. 43.17 ± 3.24 µl.min^–1^.g^–1^) and absolute sodium excretion (U_Na_V) (1.08 ± 0.27 vs, 6.38 ± 0.76 µmol.min^–1^.g^–1^). After denervation ACF TGR showed improved autoregulation of RBF: at lowest RAP level (80 mmHg) the value was higher than in innervated ACF TGR (6.92 ± 0.26 vs. 4.54 ± 0.22 ml.min^–1^.g^–1^, *p* < 0.05). Also, the pressure-natriuresis relationship was markedly improved after RDN: at the RAP of 80 mmHg UF equaled 4.31 ± 0.99 vs. 0.26 ± 0.09 µl.min^–1^.g^–1^ recorded in innervated ACF TGR, U_Na_V was 0.31 ± 0.05 vs. 0.04 ± 0.01 µmol min^–1^.g^–1^ (*p* < 0.05 in all cases). In conclusion, in our model of hypertensive rat with ACF-induced HF, RDN improved autoregulatory capacity of RBF and the pressure-natriuresis relationship when measured at the stage of HF decompensation.

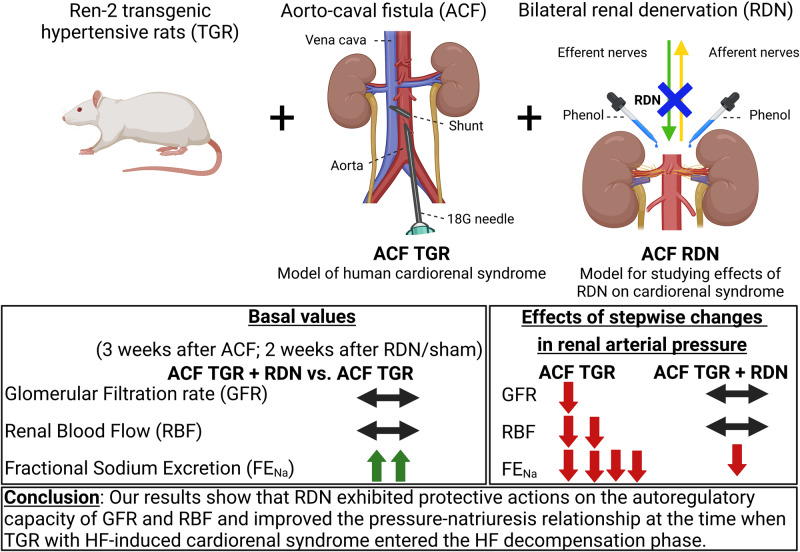

## Introduction

Heart failure (HF) is a global pandemic and almost 40% of HF patients die within 1 year of the diagnosis and 70% within 5 years, despite the availability of multiple therapeutic measures and recent pharmacological advances [1–3]. The prognosis is worst when HF is accompanied by impairment of renal function (“cardiorenal syndrome”) [2, 4–6]. Hypertension is the single most important modifiable factor for the development of HF and renal damage: it precedes HF in up to 85% of patients [7–10], especially those with “cardiorenal syndrome” [2, 4, 6, 11–13].

There is growing awareness that the “cardiorenal syndrome” reflects a bidirectional relationship between the heart and the kidney, and dysfunction of one organ might induce and/or worsen the dysfunction of the other [8–10]. It has been shown that abnormalities in renal sodium handling and alterations in renal hemodynamics are present long before HF becomes manifest [14–19]. Thus, the view has emerged that the kidney is also a “culprit” of the cardiorenal syndrome and progression of HF. Nevertheless, comprehensive understanding of the precise interacting mechanism(s) contributing to the role of kidney in the pathophysiology of HF and particularly cardiorenal syndrome has not yet been attained.

Recent research has focused on the role of sympathetic nervous system (SNS) and particularly on the application of both afferent and efferent renal denervation (RDN). There is vast evidence of the importance of the SNS in the regulation of renal function and blood pressure (BP) [20, 21] and its role in the progression of HF [20–22] and particularly in the development of the cardiorenal syndrome [2, 4–6]. RDN was originally introduced for the treatment of resistant hypertension and is now regarded a safe and effective procedure bringing long-term BP-lowering [23–25], therefore new clinical indications for its application beyond hypertension are considered [25–27]. Recent studies have suggested that RDN may be a promising tool in the treatment of HF and the cardiorenal syndrome [4, 5, 27–29]. The principal mechanism(s) of favorable effects of RDN remain unidentified and it is apparent the beneficial actions follow multiple pathways. Therefore, preclinical studies are urgently needed to elucidate mechanisms whereby RDN improves the outcome in the cardiorenal syndrome.

The rat model of volume overload induced by creation of the aorto-caval fistula (ACF) reasonably well mimics human HF [30–32]. By employing this approach in the Ren-2 renin transgenic rat (TGR), a strain that combines endogenous activation of the RAS and hypertension [33, 34], the two factors critical in the progression of HF and development of cardiorenal syndrome [4–6, 35–37], we demonstrated in our recent studies that ACF TGR represents a reasonable model of human cardiorenal syndrome [31, 38–40].

We found recently that RDN substantially attenuated HF-dependent mortality in ACF TGR, but these beneficial effects were not associated with improvement of reduced renal blood flow (RBF) in ACF TGR [39]. Our present findings on one side further support the importance of the kidney in the pathophysiology of HF, particularly of the cardiorenal syndrome and, on the other side, imply that beneficial actions of RDN cannot be simply ascribed to the removal of deleterious influence of increased renal sympathetic nerve activity (RSNA) on the renal vasculature. However, it is important to recognize that the absence of RBF improvement in ACF TGR after RDN does not unequivocally mean that RDN did not exhibit beneficial effects via some renal mechanism. Besides the basic indices of renal function, the capacity of RBF and glomerular filtration rate (GFR) autoregulation and of the renal pressure-natriuresis mechanism are also important, as indicated by the research of the past five decades. This has shown that impaired renal autoregulation and blunted pressure-natriuresis relationship play a critical role in the progression of various cardio-renal diseases [41–52].

Accordingly, the aim of the preset study was to assess autoregulation of the renal hemodynamics and the pressure-natriuresis relationship at the time point of two weeks after RDN equivalent to three weeks after creation of ACF. This time was selected because at that stage untreated innervated ACF TGR began markedly to die (i.e., exhibited an onset of decompensation phase of HF), whereas those that underwent RDN displayed 100% survival rate (i.e., were still in the compensation phase of HF) [39].

## Methods

### Ethical approval and animals

The study was performed in accordance with the guidelines and practices established by the Animal Care and Use Committee of the Institute for Clinical and Experimental Medicine (IKEM), Prague, which accord with the European Convention on Animal Protection and Guidelines on Research Animal Use, and was approved by this committee and subsequently by the Ministry of Health of the Czech Republic (the decision number for this project is 18680/2020-4/OVZ). All animals used in the study were bred in IKEM, which is accredited by the Czech Association for Accreditation of Laboratory Animals. Experiments were performed in heterozygous TGR that were generated by breeding male homozygous TGR with female homozygous transgene-negative normotensive Hannover-Sprague Dawley rats. The study was carried out in compliance with the ARRIVE (Animals in Research: Reporting In vivo Experiments) guidelines [53].

### Heart failure model, exclusion criteria and RDN technique

Eight-weeks-old male TGR were anesthetized with intraperitoneal ketamine/midazolam mixture (Calypsol, Gedeon Richter, Hungary, 160 mg/kg and Dormicum, Roche, France, 160 mg/kg). HF variant dependent on volume overload was then induced by creating ACF using needle technique. This procedure is routinely performed in our laboratory and the details were reported previously [31, 32, 38–40]. Sham-operated rats underwent an identical procedure but without creating ACF. If a technical error occurred during ACF creation procedure or pulsatile flow in the inferior vena cava could not be confirmed, suggesting flawed ACF function, animals were excluded from the study.

Standard bilateral RDN or sham-RDN procedure was performed one week after ACF or sham surgery. Under ketamine/midazolam anesthesia, both kidneys were approached through a midline abdominal incision, both renal arteries and veins were stripped of the connective tissue and all visible nerve fibers were separated from the arteries and cut close to the hilus. Then, both renal arteries were painted with 10% phenol solution in absolute alcohol in order to destroy any remaining nerve fibers. The adjacent tissues were carefully protected from exposure to phenol solution and disruption of the major lymphatic vessels in the area was avoided. Control animals underwent laparotomy and retraction of the abdominal organs, but renal vessels were not isolated or painted with phenol solution and the renal nerves were not severed; instead, the intraabdominal area was coated with a 0.9% NaCl solution. This procedure of RDN, now employed as a golden standard method of RDN, was repeatedly shown to be fully effective [39, 54–57].

Three weeks after creation of ACF, untreated (i.e., innervated) TGR were considered to be in the phase of the transition from the compensation to the decompensation phase of HF, whereas TGR that were treated by RDN were in the compensation phase of HF, as documented in our previous studies in TGR [31, 32, 38, 39, 58].

### Surgical preparation for renal functional studies

On the day of experiment, rats were anesthetized with intraperitoneal sodium thiopental (50 mg/kg, i.p.) and placed on the thermoregulated surgical table to maintain body temperature at 37 °C. Tracheostomy was performed and a PE-240 tube was inserted to maintain patent airways, and the exterior end of the tracheal cannula was placed inside a small plastic chamber into which a humidified 95% oxygen/5% carbon dioxide mixture was continuously delivered. This has been shown to improve the stability of arterial blood pressure (BP) of barbiturate-anesthetized rats [59]. Notably, even if barbiturate anesthesia exhibits some negative effects on BP, we have previously reported that the values obtained in anesthetized rats are an accurate reflection of BP values in conscious rats [34, 45]. The right jugular vein was cannulated with a PE-50 catheter for fluid infusion and for administration of the anesthetic. PE-50 cannulas were placed in the left carotid artery and in the left femoral artery for continuous measurement of arterial BP above and below the left renal artery. Mean arterial pressure (MAP) was monitored with a pressure transducer (model MLT1050, ADInstruments) and recorded using a computerized data-acquisition system (Power Laboratory/4SP, ADInstruments). The left kidney was exposed via a flank incision, isolated from the surrounding tissue and placed in a lucite cup. The ureter was then cannulated with a PE-10 catheter. Two aortic clamps were placed on the aorta, one above the superior mesenteric artery and one below the left renal artery, to allow manipulation of renal arterial pressure (RAP). The ultrasonic transient-time flow probe (1RB, Transonic System) connected to a Transonic flowmeter was placed around the left renal artery and RBF was recorded using a computerized data-acquisition system. At the end of experiment, zero value was established by complete occlusion of the aorta. During and after the surgery, isotonic saline solution containing bovine serum albumin (6%, Sigma Aldrich Chemical Co., Prague, Czech Republic) was infused at 60 µl/min. This general surgical preparation is based on the original methods developed by Roman and Cowley for studying pressure-natriuresis in the rat [42] and modified by Wang et al. [43]. The procedure has been standardly used in our laboratory in various hypertension models [40, 43–47].

After completion of the surgical procedure, 50-min equilibration was allowed for rats to reach a steady state before initiating one 30-min control urine collection at the baseline level of RAP. Subsequently, using aortic clamps, RAP was increased or reduced to the levels as indicated for different experimental protocols (see below and as summarized in Table 1). Moreover, corresponding urine collection periods were done.

**Table 1:** Experimental groups and the values of renal arterial pressure (RAP) during individual urine collection periods (U)

RAP (mmHg)					
EXPERIMENTAL GROUP					
	U1	U2	U3	U4	U5
Innervated sham-operated TGR	Basal RAP	125	100	90	80
Sham-operated TGR + RDN	Basal RAP	125	100	90	80
Innervated ACF TGR	Basal RAP	100	90	80	125
ACF TGR + RDN	Basal RAP	100	90	80	125

*TGR* Ren-2 renin transgenic rats, *ACF* aorto-caval fistula, *RDN* renal denervation

indicates reduction of renal arterial pressure in the appropriate experimental group and in the given urine collection period

indicates increase of renal arterial pressure in the appropriate experimental group and in the given urine collection period

Urine volume was measured gravimetrically. The urinary sodium concentration was determined by flame photometry and FITC-inulin concentrations were assessed by fluorescence. FITC-inulin clearance served as a marker of GFR; the values were calculated per gram kidney weight and standard formulas were used to calculate fractional sodium excretion. At the end of the experiment, whole heart weight (HW) and then left ventricle weight (LVW) (including septum), right ventricle weight (RVW) and lung weight (“wet lung weight”) were assessed as described in our previous studies [31, 32, 38, 39, 58].

### Detailed experimental design

#### Control protocol in sham-operated and ACF TGR

In all groups in which control protocol was applied, after initial control urine collection at the basal level of RAP, additional four 30-min urine collections at the same RAP were performed.

#### Experimental protocol in sham-operated TGR

After initial control urine collection at the basal level of RAP, four 30-min urine collections were performed at RAP reduced to 125, 100, 90 and 80 mmHg. The reductions in RAP were performed sequentially from 125 to 80 mmHg.

#### Experimental protocol in ACF TGR

After initial control urine collection at the basal level of RAP, three 30-min urine collections were performed at RAP sequentially reduced to 100, 90 and 80, followed by one 30-min urine collection at RAP increased to 125 mmHg.

**Fig. 1:**
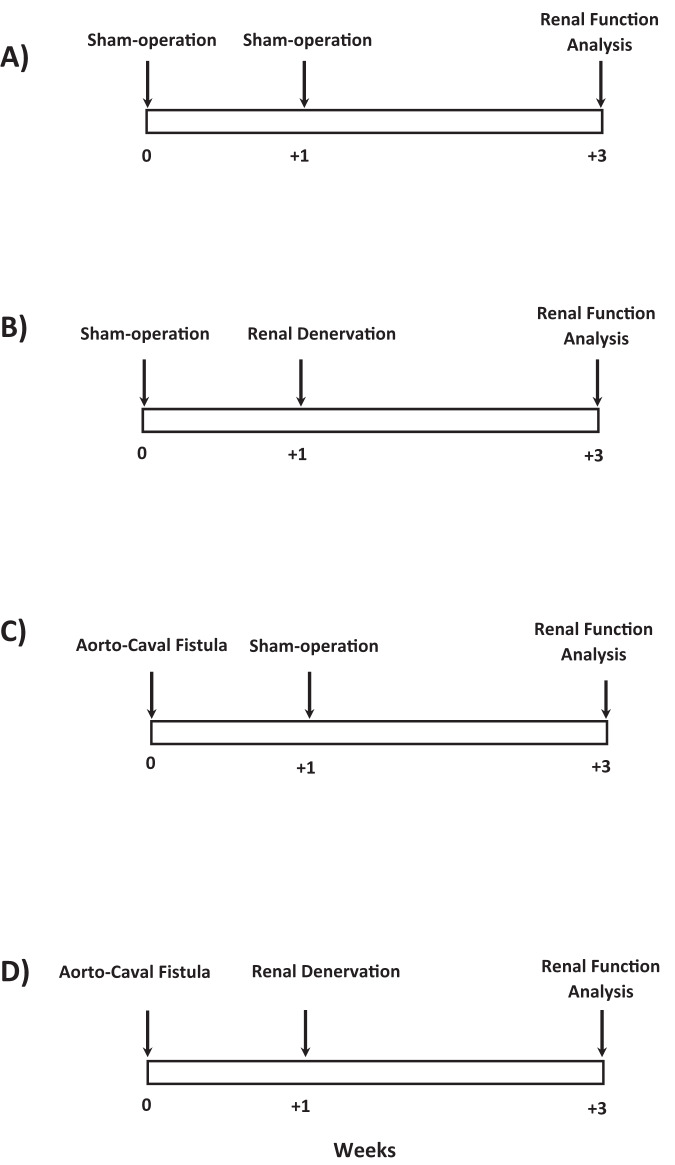
The experimental design of the whole study, delineating the time sequence of experimental maneuvers

The values of RAP used were chosen based on our previous studies employing sham-operated and ACF TGR [38–40]. Noteworthily, basal values of RAP (equivalent to MAP) are markedly lower in ACF TGR than in sham-operated TGR. Therefore, in ACF TGR groups sequential reductions to 100, 90 and 80 mmHg were first performed and then in the fifth period RAP was increased to 125 mmHg, the value which corresponds to that in the second period of sham-operated TGR groups. The aim of such experimental design was to obtain RBF, GFR, urine flow and renal sodium excretion values as far as possible comparable between the groups of the same RAP. With this approach, we were able to compare the measured values at the same RAP levels: 125, 100, 90 and 80 mmHg. An exception was the first period performed at the basal level of RAP. The experimental protocols in all experimental groups are outlined in Fig. 1 and Table 1.

The following groups were examined:

1. Sham-operated TGR + sham RDN + control protocol (*n* = 12)
2. Sham-operated TGR + sham RDN + experimental protocol (*n* = 14)
  The experimental protocol for those groups is delineated in Fig. 1A and these groups will be presented in figures and tables as “Innervated sham-operated TGR”, where “sham operated TGR” denotes rats after sham ACF creation.
3. Sham-operated TGR + RDN + control protocol (*n* = 12)
4. Sham-operated TGR + RDN + experimental protocol (*n* = 14)
  The experimental protocol for those groups is delineated in Fig. 1B and these groups will be presented in figures and tables as “Sham-operated TGR + RDN”.
5. ACF TGR + sham RDN + control protocol (*n* = 12)
6. ACF TGR + sham RDN + experimental protocol (*n* = 14)
  The experimental protocol for those groups is delineated in Fig. 1C and these groups will be presented in figures and tables as “Innervated ACF TGR”.
7. ACF TGR + RDN + control protocol (*n* = 12)
8. ACF TGR + RDN + experimental protocol (*n* = 14)

The experimental protocol for those groups is delineated in Fig. 1D and these groups will be presented in figures and tables as “ACF TGR + RDN”.

### Histological evaluation of the heart and kidney tissues

In separate appropriately matched four experimental groups (*n* = 9 in each), i.e., innervated sham-operated TGR, sham-operated TGR + RDN, innervated ACF TGR and ACF TGR + RDN, the hearts and kidneys were subjected to histological examination of the myocardium and renal cortex as described previously [57].

#### Heart tissue

The rats were anesthetized with a combination of midazolam 50 mg kg^–1^ (Dormicum, Roche Ltd., Prague, Czech Republic) and ketamine 50 mg.kg^–1^ (Calypsol, Gedeon Richter Ltd., Budapest, Hungry) i.p. The beating (pulsating) organ i.e., the native heart was perfused in situ with 20 ml of Thomas cardioplegia solution and subsequently fixed in 4% paraformaldehyde in phosphate-buffered saline and embedded into Tissue-Tek. The blocks were cut using a cryo-microtome, and cardiomyocyte width was measured in the subendocardium, mid-myocardium, and subepicardium of the left ventricle (LV). Cardiomyocyte length was measured only in the midmyocardium; in each layer 30 cardiomyocytes were assessed. To avoid underestimation, only the cells in which the nucleus was visible were measured. Since there were no significant differences in the cardiomyocyte width between the layers, the data from the subendocardium, midmyocardium, and subepicardium were pooled, as was also practiced by other investigators [60]. Analysis of LV fibrosis was performed in sections stained with Picrosirius red (Direct Red 80, Sigma Aldrich, MO, USA) as described in detail previously [61]. The interstitial collagen was analyzed in polarized light using 10 images of the LV scanned from a midmyocardium, without perivascular areas (magnification 200x, microscope Nikon eclipse Ni-E, camera Nikon DS-L3, Tokyo, Japan). The percent area of myocardial fibrosis was calculated semiquantitatively, using imaging software NIS-Elements Ar (LIM, Prague, Czech Republic).

#### Kidney tissue

The kidneys were used to assess glomerular damage and tubulointerstitial injury. The kidneys were fixed in 4% formaldehyde, dehydrated and embedded in paraffin. The sections stained with periodic acid, for Schiff reaction, were examined and evaluated in a blind-test fashion. Fifty glomeruli in each kidney were examined on a semi-quantitative scale. The evaluation was as follows: *grade 0*, all glomeruli normal; *grade 1*, sclerotic area up to 25% (minimal sclerosis); *grade 2*, sclerotic area 25 to 50% (moderate sclerosis); *grade 3*, sclerotic area 50 to 75% (moderate-to-severe sclerosis); *grade 4*, sclerotic area 75 to 100% (severe sclerosis). The glomerulosclerosis index (GSI) was calculated using the following formula: GSI = [(1 x n_1_) + (2 x n_2_) + (3 x n_3_) + (4 x n_4_)]/(n_0_ + n_1_ + n_2_ + n_3_ + n_4_), where n_x_ is the number of glomeruli in each grade of glomerulosclerosis. Kidney cortical tubulointerstitial injury was evaluated as defined by Nakano et al. [47], to determine inflammatory cell infiltration, tubular dilatation, atrophy, or interstitial fibrosis. The injury was graded semi-quantitatively using the following scale of lesions: grade 0, no abnormal findings; 1, mild (<25% of the cortex); 2, moderate (25–50% of the cortex); 3, severe (>50% of the cortex). The lesions were assessed in at least 30 random and non-overlapping fields in the renal cortex. Thus, the maximum score for GSI is 4 and for the index of kidney tubulointerstitial injury (TSI) is 3. The values of GSI < 0.5 and TSI < 0.4 are considered as healthy renal tissue without sign of significant renal damage. This method is always employed in our studies evaluating the degree of kidney damage [57, 62, 63].

### Statistical Analysis

All values are expressed as means ± SEM. Using the Graph-Pad Prism software (Graph Pad Software, San Diego, CA, USA), statistical analysis was performed: Student´s *t*-test for unpaired data, or one-way analysis of variance (ANOVA) followed by Tukey-Kramer multiple comparisons test when appropriate. ANOVA for repeated measurements was performed for the analysis within groups (e.g., for the analysis of autoregulatory capacity of RBF). Values exceeding 95% probability limits (*p* < 0.05, two-sided) were considered statistically significant.

## Results

As summarized in Table 2, in sham-operated TGR the denervation resulted in significant decreases in MAP when compared with their innervated counterparts. ACF creation resulted in significant decreases in MAP, but RDN did not elicit statistically significant decreases in MAP in ACF TGR as compared with innervated ACF TGR. Three weeks after ACF creation TGR displayed prominent bilateral cardiac hypertrophy and, as seen from the increases in the ratio of RVW to LVW, the right ventricle (RV) hypertrophy was more distinct than that of the left ventricle (LV). Moreover, innervated ACF TGR displayed significantly greater lung weight as compared with innervated sham-operated TGR. RDN did not alter any organ parameters in sham-operated TGR. In contrast, in ACF TGR group RDN significantly reduced whole HW, LVW, RVW and lung weight as compared with the corresponding values in their innervated counterparts. There were no significant differences in basal GFR between experimental groups. Basal RBF was significantly lower in innervated ACF TGR than in innervated sham-operated TGR and RDN did not significantly alter it in any group. Renal vascular resistance (RVR) was significantly higher in innervated ACF TGR as compared with innervated sham-operated TGR, and RDN decreased RVR in sham-operated TGR as well as in ACF TGR. Basal urine flow, and absolute and fractional sodium excretion were markedly reduced in innervated ACF TGR as compared with innervated sham-operated TGR, and RDN did not alter these parameters in sham-operated TGR, but significantly increased them in ACF TGR (the exact values for each parameter are provided in Table 2 and therefore are not presented in the text).

**Table 2:** Basal values of arterial blood pressure, renal function and organ weights pooled from groups undergoing control and experimental protocols

	Group			
	Innervated	Sham-operated TGR	Innervated	ACF TGR
	sham-operated TGR	+RDN	ACF TGR	+RDN
	**(*n*** = **26)**	**(*n*** = **26)**	**(*n*** = **26)**	**(*n*** = **26)**
Body weight (g)	420 ± 5	419 ± 6	418 ± 5	404 ± 6
Mean arterial pressure (mmHg) (in a. femoralis)	160 ± 3	133 ± 2^*^	115 ± 2	108 ± 3^**@**^
Renal blood flow (ml.min^–1^.g^–1^)	10.87 ± 0.38	12.17 ± 0.38	6.91 ± 0.33	8.41 ± 0.38^**@**^
Renal vascular resistance (mmHg.ml^–1^.min^–1^.g^–1^)	14.60 ± 0.62	11.37 ± 0.31^*^	15.72 ± 0.84	12.67 ± 0.66^**#**^
Glomerular filtration rate (ml.min^–1^.g^–1^)	0.946 ± 0.074	1.075 ± 0.14	1.029 ± 0.15	1.155 ± 0.14
Filtration Fraction (%)	15.8 ± 1.02	15.2 ± 0.96	29.1 ± 1.12	26.1 ± 1.06^**@**^
Urine flow (µl.min^–1^.g^–1^)	43.17 ± 3.24	41.93 ± 3.64	11.30 ± 1.79	28.65 ± 3.12^**#@**^
Absolute sodium excretion (µmol. min^–1^.g^–1^)	6.38 ± 0.76	5.87 ± 0.53	1.08 ± 0.27	3.54 ± 0.45^**#@**^
Fractional sodium excretion (%)	5.80 ± 0.52	4.53 ± 0.73	0.36 ± 0.11	2.04 ± 0.26^**#@**^
Heart weight (mg)/Body weight (g)	3.67 ± 0.04	3.55 ± 0.03	5.55 ± 0.11	4.49 ± 0.09^**#@**^
Left ventricle weight (mg)/Body weight (g)	2.83 ± 0.03	2.76 ± 0.03	3.53 ± 0.05	3.14 ± 0.05^**#@**^
Right ventricle weight (mg)/Body weight (g)	0.493 ± 0.01	0.476 ± 0.009	1.143 ± 0.038	0.845 ± 0.024^**#@**^
Right ventricle weight (mg)/Left ventricle weight (mg)	0.176 ± 0.003	0.174 ± 0.004	0.327 ± 0.009	0.273 ± 0.005^**#@**^
Lung weight (mg)/Body weight (g)	4.027 ± 0.06	4.357 ± 0.07	7.687 ± 0.11	5.661 ± 0.0.09^**#@**^
Liver weight (mg)/ Body weight (g)	39.52 ± 0.31	38.14 ± 0.49	38.48 ± 0.47	38.69 ± 0.44
Kidney weight (mg)/Body weight (g)	3.97 ± 0.02	3.93 ± 0.03	3.91 ± 0.03	3.92 ± 0.03

The values are the means ± SEM. TGR, Ren-2 renin transgenic rats; *ACF* aorto-caval fistula, *RDN* renal denervation. ^*^p < 0.05 Sham-operated TGR + RDN vs. Innervated sham-operated TGR, ^**#**^*p* < 0.05 ACF TGR + RDN vs. Innervated ACF TGR, ^**@**^*p* < 0.05 ACF TGR + RDN vs. Sham-operated TGR + RDN

**Fig. 2:**
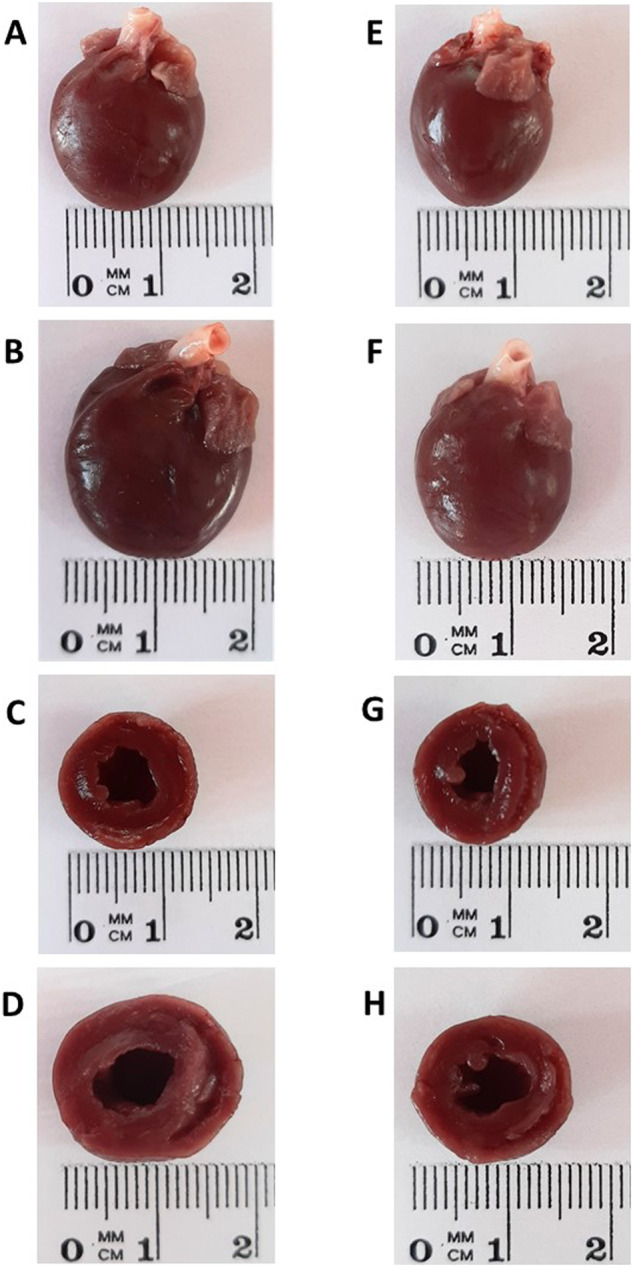
Representative images of the whole heart and transversely cut heart in innervated [i.e., without renal denervation (RDN)] sham-operated heterozygous Ren-2 transgenic rats (TGR) (**A**, **C**) (average whole heart weight in this group is 1542 ± 26 mg), innervated TGR with aorto-caval fistula (ACF) (**B**, **D**) (average whole heart weight in this group is 2321 ± 43 mg), sham-operated TGR after RDN (**E**, **G**) (average whole heart weight in this group is 1501 ± 28 mg) and ACF TGR after RDN (**F**, **H**) (average whole heart weight in this group is 1811 ± 23 mg)

Gross morphological changes in the heart are shown in Fig. 2, which shows representative whole heart images in innervated sham-operated TGR, sham-operated TGR + RDN, innervated ACF TGR and ACF TGR + RDN groups, as well as the images of transversally cut hearts. These images show that innervated sham-operated TGR exhibit signs of concentric LV hypertrophy and ACF creation caused dilatation of the LV as well as RV cavities with further increase of wall mass.

**Fig. 3:**
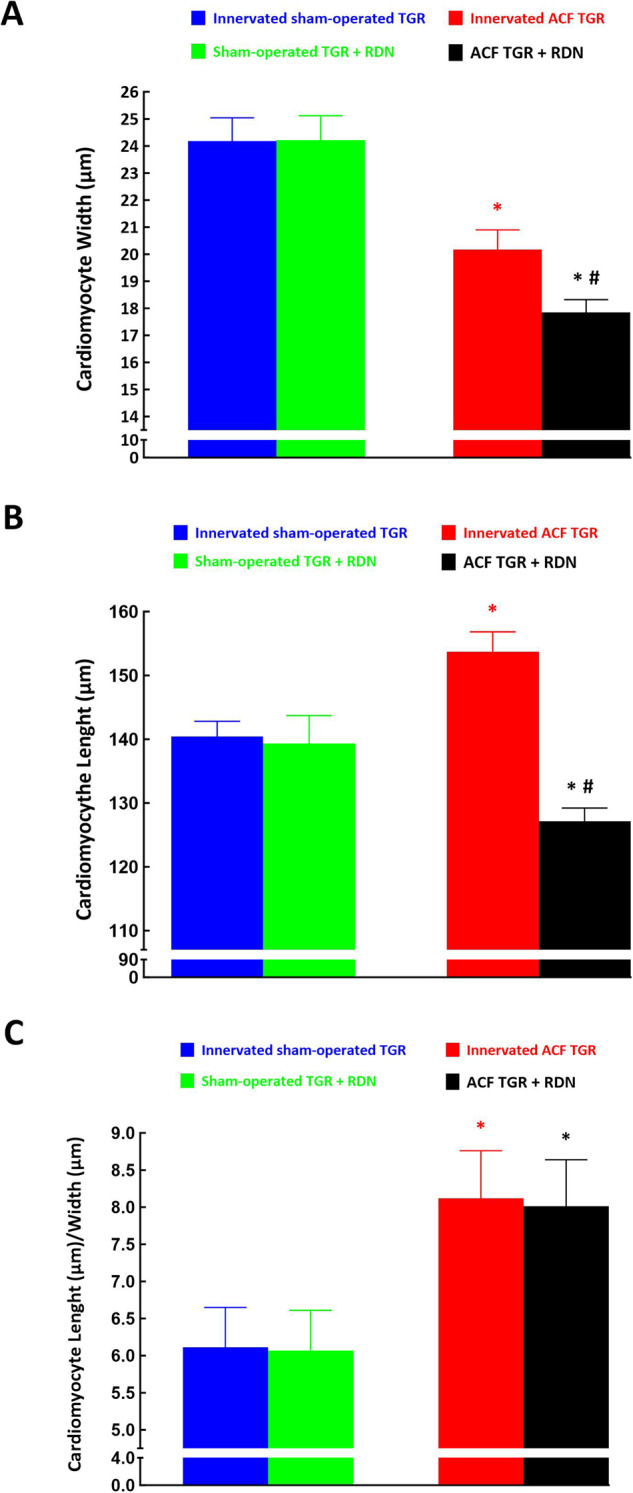
Cardiomyocyte width (**A**), cardiomyocyte length (**B**), the ratio of cardiomyocyte length to width (**C**) in innervated [i.e., without renal denervation (RDN)] sham-operated Ren-2 transgenic rats (TGR) (blue bars), in sham-operated TGR that underwent RDN (green bars), innervated TGR with aorto-caval fistula (ACF) (red bars) and ACF TGR with RDN (black bars). ^*^p < 0.05 compared with the data for sham-operated counterparts. ^#^*p* < 0.05 ACF TGR + RDN compared with innervated ACF TGR

Figure 3 summarizes the morphological changes at the cardiomyocyte level. Innervated ACF TGR showed lower cardiomyocyte width as compared with innervated sham-operated TGR (20.17 ± 0.73 vs. 24.18 ± 0.86 µm, *p* < 0.05), whereas innervated ACF TGR showed higher cardiomyocyte length as compared with innervated sham-operated TGR (153.7 ± 3.13 vs. 177.1 ± 2.11 µm, *p* < 0.05); this resulted in higher ratio of cardiomyocyte length to cardiomyocyte width in innervated ACF TGR as compared with innervated sham-operated TGR (8.12 ± 0.72 vs. 6.11 ± 0.61 µm, *p* < 0.05). RDN did not alter the cardiomyocyte width or cardiomyocyte length in sham-operated TGR but, in contrast, it caused significant and proportional decreases in cardiomyocyte width (Fig. 3A) and cardiomyocyte length (Fig. 3B) in ACF TGR, which resulted in the maintenance of increased ratio of cardiomyocyte length to cardiomyocyte width than observed in innervated sham-operated TGR (Fig. 3C). These findings at the cardiomyocyte level corroborate the observation from whole organs (Fig. 2) that both innervated and denervated ACF TGR show signs of eccentric LV hypertrophy.

There were no significant differences in the myocardial fibrosis (expressed in %) in the LV between innervated sham-operated TGR and innervated ACF TGR (3.50 ± 0.49 vs. 3.81 ± 0.46%) and RDN did not significantly alter myocardial fibrosis in the LV in sham-operated TGR (3.37 ± 0.58%) or in ACF TGR (4.17 ± 0.67%). There were no signs of myocardial fibrosis or appearance of scar tissue in any experimental groups. Representative histological images of myocardial fibrosis in the LV are shown in Supplementary Fig. 1. There were no significant differences in GSI between innervated sham-operated TGR and innervated ACF TGR (0.26 ± 0.12 vs. 0.18 ± 0.06) and RDN did not change GSI, similarly in sham-operated TGR (0.22 ± 0.06) and in ACF TGR (0.24 ± 0.04). Noteworthily, all values for GSI were very low, indicating no significant renal damage in any group: the index value did not exceed 0.5, (the value considered as a borderline between healthy and renal damage) in any group or in any individual animal. The same is valid for TSI (data not shown). Representative images of renal parenchyma are shown in Supplementary Fig. 2.

**Fig. 4:**
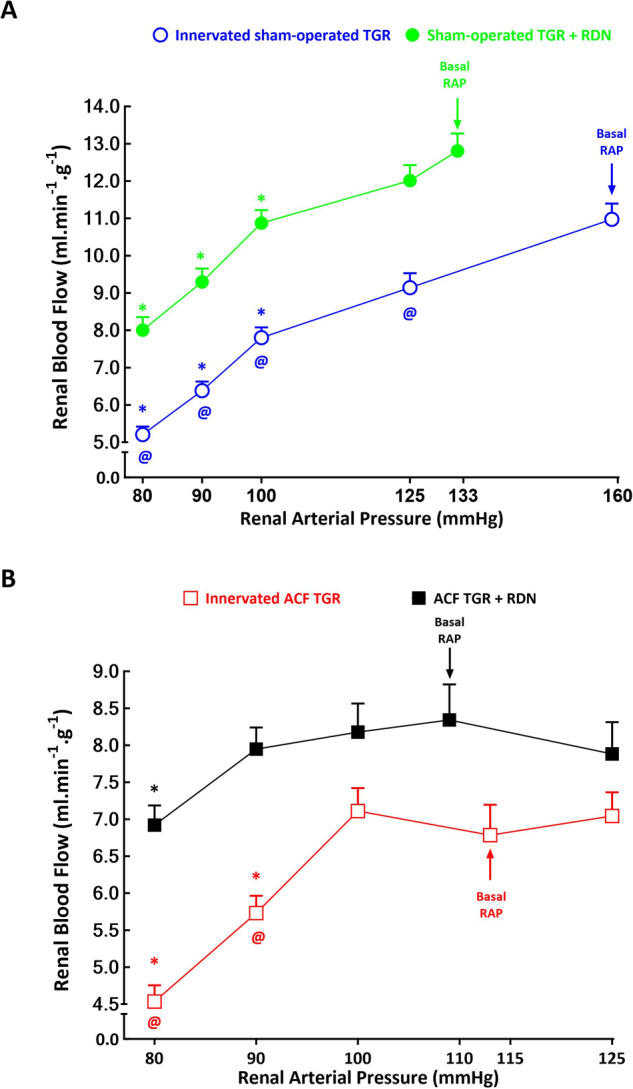
Relationship between renal arterial pressure (RAP) and renal blood flow in innervated [i.e., without renal denervation (RDN)] sham-operated Ren-2 transgenic rats (TGR) (open blue circles connected with blue line), in sham-operated TGR that underwent RDN (closed green circles connected with green line) (**A**), innervated TGR with aorto-caval fistula (ACF) (open red squares connected with red line) and ACF TGR that underwent RDN (closed black squares connected with black line) (**B**) in animals exposed to experimental protocol. ^*^p < 0.05 compared with values at the basal RAP, ^**@**^*p* < 0.05 compared with the corresponding values at the same RAP. Symbols are always shown in the color appropriate for the corresponding experimental group

**Fig. 5:**
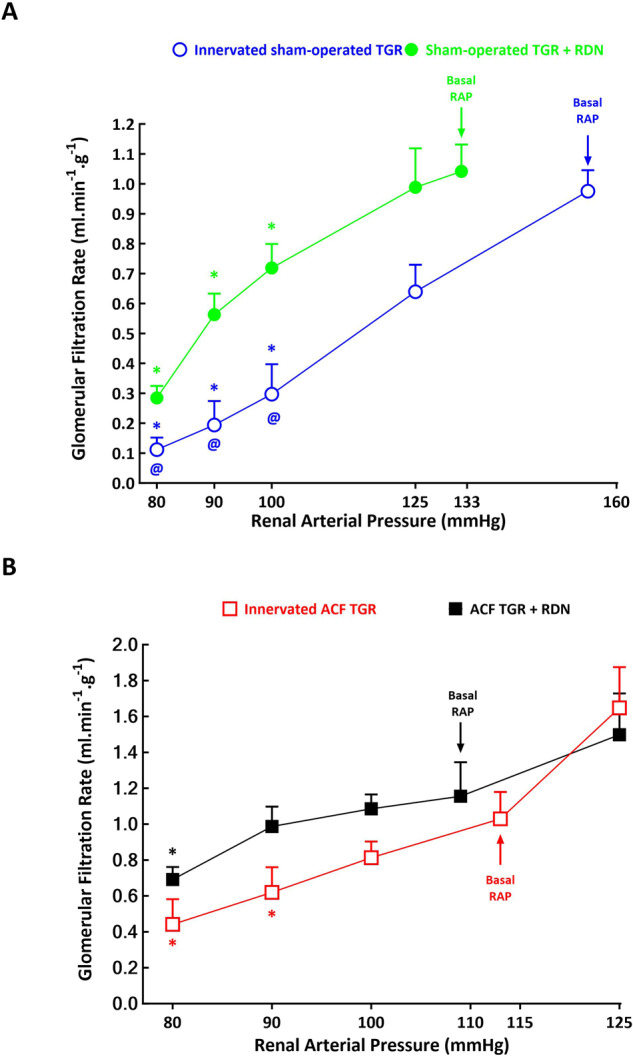
Relationship between renal arterial pressure (RAP) and glomerular filtration rate in innervated [i.e., without renal denervation (RDN)] sham-operated Ren-2 transgenic rats (TGR) (open blue circles connected with blue line), in sham-operated TGR that underwent RDN (closed green circles connected with green line) (**A**), innervated TGR with aorto-caval fistula (ACF) (open red squares connected with red line) and ACF TGR that underwent RDN (closed black squares connected with black line) (**B**) in animals exposed to experimental protocol. ^*^p < 0.05 compared with values at the basal RAP, ^**@**^*p* < 0.05 compared with corresponding values at the same RAP. Symbols are always shown in the color appropriate for the corresponding experimental group

**Fig. 6:**
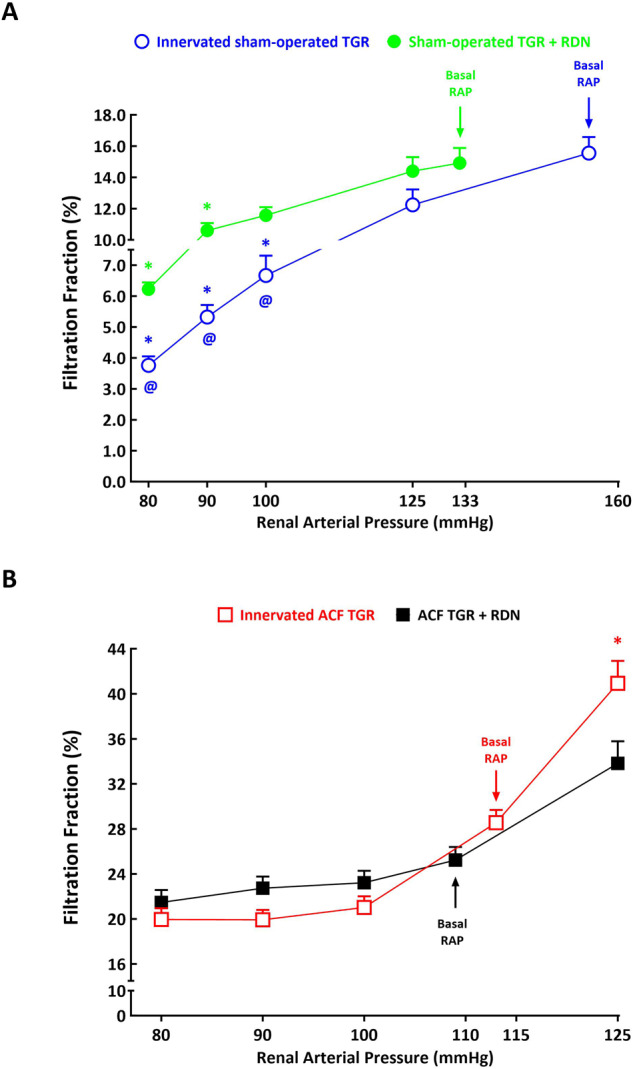
Relationship between renal arterial pressure (RAP) and filtration fraction in innervated [i.e., without renal denervation (RDN)] sham-operated Ren-2 transgenic rats (TGR) (open blue circles connected with blue line), in sham-operated TGR that underwent RDN (closed green circles connected with green line) (**A**), innervated TGR with aorto-caval fistula (ACF) (open red squares connected with red line) and ACF TGR that underwent RDN (closed black squares connected with black line) (**B**) in animals exposed to experimental protocol. ^*^p < 0.05 compared with values at the basal RAP, ^**@**^*p* < 0.05 compared with corresponding values at the same RAP. Symbols are always shown in the color appropriate for the corresponding experimental group

As shown in Figs. 4A and 5A, innervated sham-operated TGR exhibited impaired autoregulatory capacity of RBF and GFR in response to reduction of RAP, with profound decreases in RBF and particularly GFR at the RAP of 100 mmHg and lower. Remarkably, RBF decreased from 10.98 ± 0.42 ml.min^–1^.g^–1^ at the basal RAP to 5.21 ± 0.21 ml.min^–1^.g^–1^ at 80 mmHg, (*p* < 0.05) and, respectively, GFR decreased from 0.98 ± 0.13 ml.min^–1^.g^–1^ to 0.12 ± 0.04 ml.min^–1^.g^–1^ (*p* < 0.05). These responses in RBF and GFR occurred in parallel with significant profound decreases in filtration fraction (FF): from 15.6 ± 1.1% to 3.8 ± 0.3%, respectively (*p* < 0.05) (Fig. 6A).

In sham-operated TGR denervation did not prevent the decrease in RBF and GFR in response to reductions in RAP, but the absolute values of RBF and GFR at 100 mmHg and lower were significantly higher than in innervated sham-operated TGR: at the lowest RAP (80 mmHg) RBF in denervated sham-operated TGR was 8.01 ± 0.35 ml.min^–1^.g^–1^ and in innervated sham-operated TGR it was 5.21 ± 0.21 ml.min^–1^.g^–1^, i.e., significantly lower (*p* < 0.05) (Fig. 4A). Also, the decreases in FF were first noticed at 90 mmHg and were less prominent (Figs. 4A, 5A and 6A).

As shown in Figs. 4B and 5B, in innervated ACF TGR the RAP reductions from basal values to 100 mmHg or an increase to 125 mmHg did not significantly affect RBF and GFR, but further reduction to 90 and to 80 mmHg elicited significant decreases in RBF: it decreased from the basal of 6.79 ± 0.41 ml.min^−1^.g^−1^ to 4.54 ± 0.22 ml.min^–1^.g^−1^ at 80 mmHg (*p* < 0.05, Fig. 4B) and, respectively, GFR decreased from the 1.03 ± 0.15 ml.min^–1^.g^–1^ to 0.45 ± 0.15 ml.min–^1^.g^–1^ (*p* < 0.05, Fig. 5B), without significant alterations in filtration fraction (Fig. 6B).

As shown in Figs. 4B and 5B, in ACF TGR denervation improved autoregulatory capacity of RBF and GFR: reduction of RAP to 90 mmHg did not elicit significant decreases and further reduction to 80 mmHg caused only slight decreases as compared with basal values. This was particularly evident in the case of RBF: in denervated ACF TGR it was significantly higher than in innervated ACF TGR at the RAP of 90 mmHg (7.95 ± 0.29 vs 5.73 ± 0.23 ml.min^–1^.g^–1^, *p* < 0.05) as well as at 80 mmHg (6.92 ± 0.26 vs 4.54 ± 0.22 ml.min^–1^.g^–1^, *p* < 0.05) (Fig. 4B).

**Fig. 7:**
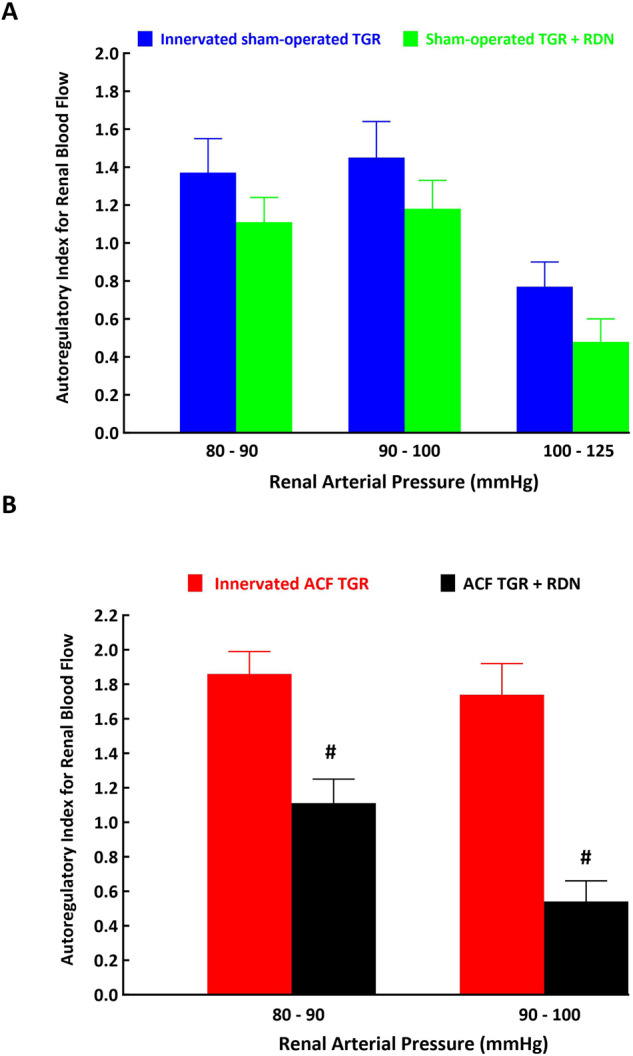
Autoregulation index values (Semple and de Wardener, reference #65) for renal blood flow responses to reductions in renal arterial pressure from 125 to 100 mmHg, from 100 to 90 mmHg and from 90 to 80 mmHg in innervated [i.e., without renal denervation (RDN)] sham-operated Ren-2 transgenic rats (TGR) (blue bars), in sham-operated TGR that underwent RDN (green bars) (**A**), innervated TGR with aorto-caval fistula (ACF) (red bars) and ACF TGR with RDN (black bars) (**B**). ^#^*p* < 0.05 ACF TGR + RDN compared with innervated ACF TGR

Figure 7 helps to analyse the autoregulatory capacity of RBF using the autoregulation index (AI) calculated according to the method of Semple and de Wardener [64]. Evidently, in sham-operated TGR denervation did not improve this capacity following reductions of RAP (Fig. 7A). In contrast, RDN markedly improved RBF autoregulatory capacity in ACF TGR as compared with innervated ACF TGR following reductions of RAP from 100 to 90 mmHg (0.54 ± 0.12 vs. 1.74 ± 0.18, *p* < 0.05) and from 90 to 80 mmHg in ACF TGR (1.11 ± 0.14 vs. 1.86 ± 0.13, *p* < 0.05) (Fig. 7B).

**Fig. 8:**
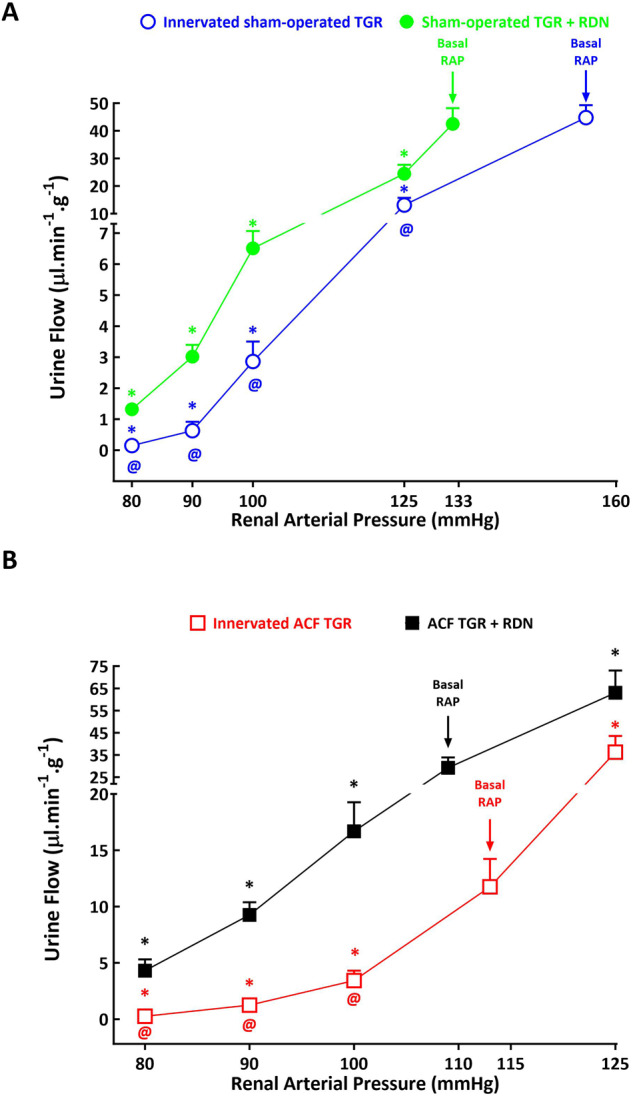
Relationship between renal arterial pressure (RAP) and urine flow in innervated [i.e., without renal denervation (RDN)] sham-operated Ren-2 transgenic rats (TGR) (open blue circles connected with blue line), in sham-operated TGR that underwent RDN (closed green circles connected with green line) (**A**), innervated TGR with aorto-caval fistula (ACF) (open red squares connected with red line) and ACF TGR that underwent RDN (closed black squares connected with black line) (**B**) in animals exposed to experimental protocol. ^*^p < 0.05 compared with values at the basal RAP, ^**@**^*p* < 0.05 compared with corresponding values at the same RAP. Symbols are always shown in the color appropriate for the corresponding experimental group

**Fig. 9:**
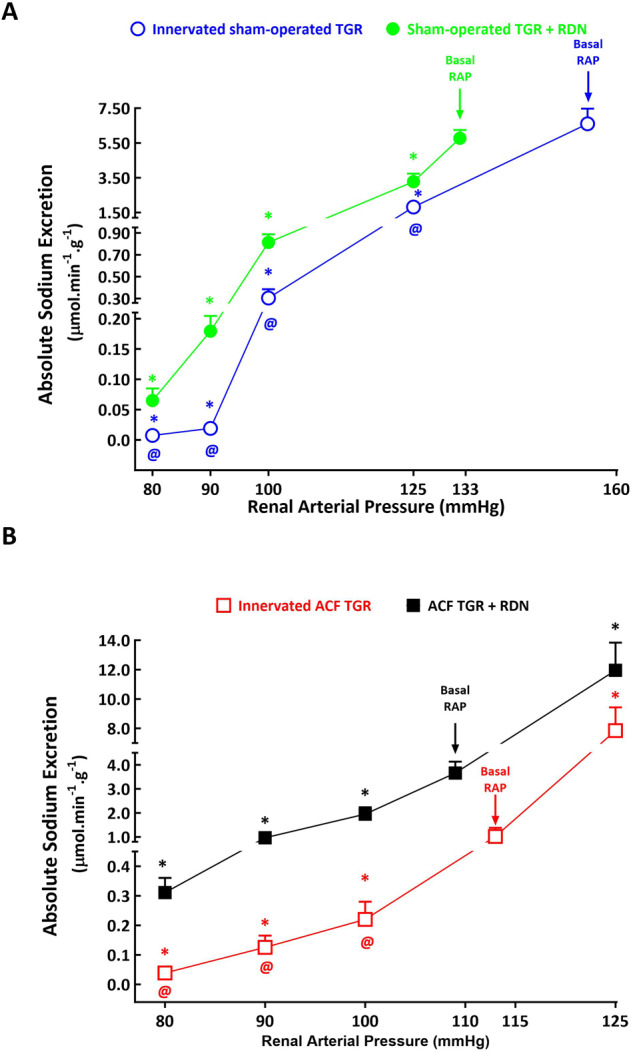
Relationship between renal arterial pressure (RAP) and absolute sodium excretion in innervated [i.e., without renal denervation (RDN)] sham-operated Ren-2 transgenic rats (TGR) (open blue circles connected with blue line), in sham-operated TGR that underwent RDN (closed green circles connected with green line) (**A**), innervated TGR with aorto-caval fistula (ACF) (open red squares connected with red line) and ACF TGR that underwent RDN (closed black squares connected with black line) (**B**) in animals exposed to experimental protocol. ^*^p < 0.05 compared with values at the basal RAP, ^**@**^*p* < 0.05 compared with corresponding values at the same RAP. Symbols are always shown in the color appropriate for the corresponding experimental group

**Fig. 10:**
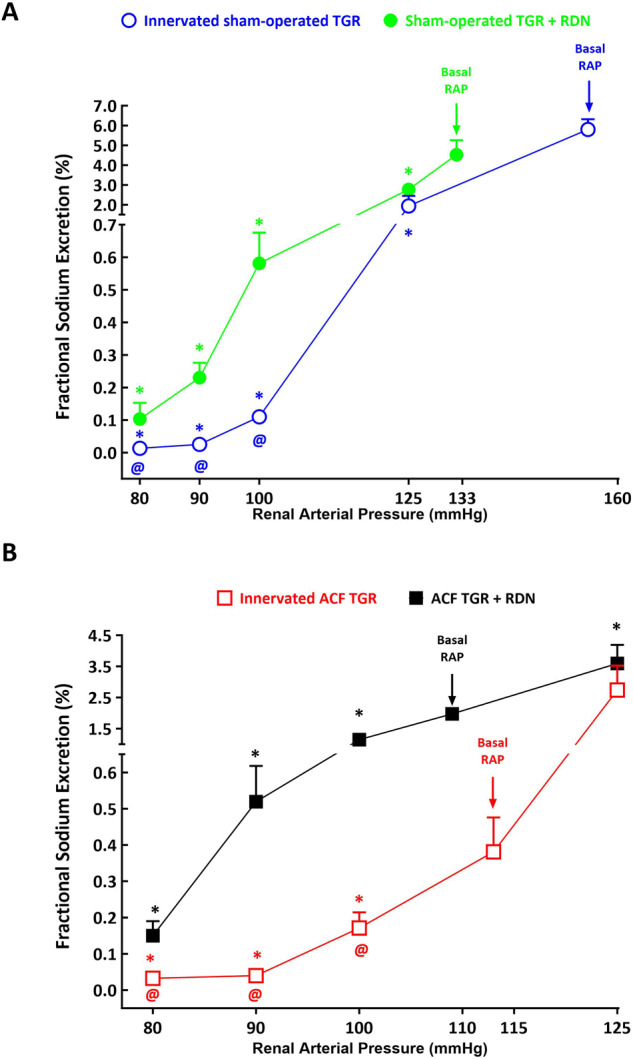
Relationship between renal arterial pressure (RAP) and fractional sodium excretion in innervated [i.e., without renal denervation (RDN)] sham-operated Ren-2 transgenic rats (TGR) (open blue circles connected with blue line), in sham-operated TGR that underwent RDN (closed green circles connected with green line) (**A**), innervated TGR with aorto-caval fistula (ACF) (open red squares connected with red line) and ACF TGR that underwent RDN (closed black squares connected with black line) (**B**) in animals exposed to experimental protocol. ^*^p < 0.05 compared with values at the basal RAP, ^**@**^*p* < 0.05 compared with corresponding values at the same RAP. Symbols are always shown in the color appropriate for the corresponding experimental group

As shown in Figs. 8A, 9A and 10A, urine flow and absolute and fractional sodium excretion at basal level of RAP did not significantly differ between innervated and denervated sham-operated TGR. The reductions in RAP resulted in significant decreases in these three parameters in innervated as well as in denervated sham-operated TGR, however, the decreases were significantly greater in innervated sham-operated TGR than their denervated counterparts. This was particularly evident at lower levels of RAP: at 80 mmHg denervated sham-operated TGR showed higher urine flow (1.32 ± 0.15 vs. 0.15 ± 0.14, µl.min^–1^.g^–1^, *p* < 0.05), absolute sodium excretion (0.065 ± 0.019 vs. 0.007 ± 0.002, µmol.min^–1^.g^–1^, *p* < 0.05) and fractional sodium excretion (0.11 ± 0.05 vs. 0.014 ± 0.003%, *p* < 0.05) than observed in innervated sham-operated TGR (Figs. 8A, 9A and 10A).

As shown in Figs. 8B, 9B and 10B, urine flow and the absolute and fractional sodium excretion were at the basal level of RAP significantly lower in innervated ACF TGR as compared with denervated ACF TGR (the relevant values are given in Table 2). There were no significant differences in urine flow and absolute and fractional sodium excretion between innervated and denervated ACF TGR when RAP was elevated to 125 mmHg. In both groups reduction of RAP caused significant decreases in urine flow and absolute and fractional sodium excretion, however, the decreases in denervated ACF TGR were significantly attenuated as compared with the data observed in innervated ACF TGR. This was particularly evident at lower levels of RAP (100, 90 and 80 mmHg): at 80 mmHg denervated ACF TGR showed higher urine flow (4.31 ± 0.0.99 vs. 0.15 ± 0.03, µl.min^–1^.g^–1^, *p* < 0.05), absolute sodium excretion (0.31 ± 0.05 vs. 0.04 ± 0.01, µmol.min^–1^.g^–1^, *p* < 0.05) and fractional sodium excretion (0.15 ± 0.04 vs. 0.03 ± 0.01%, *p* < 0.05) as compared with innervated ACF TGR (Figs. 8B, 9B and 10B).

**Fig. 11:**
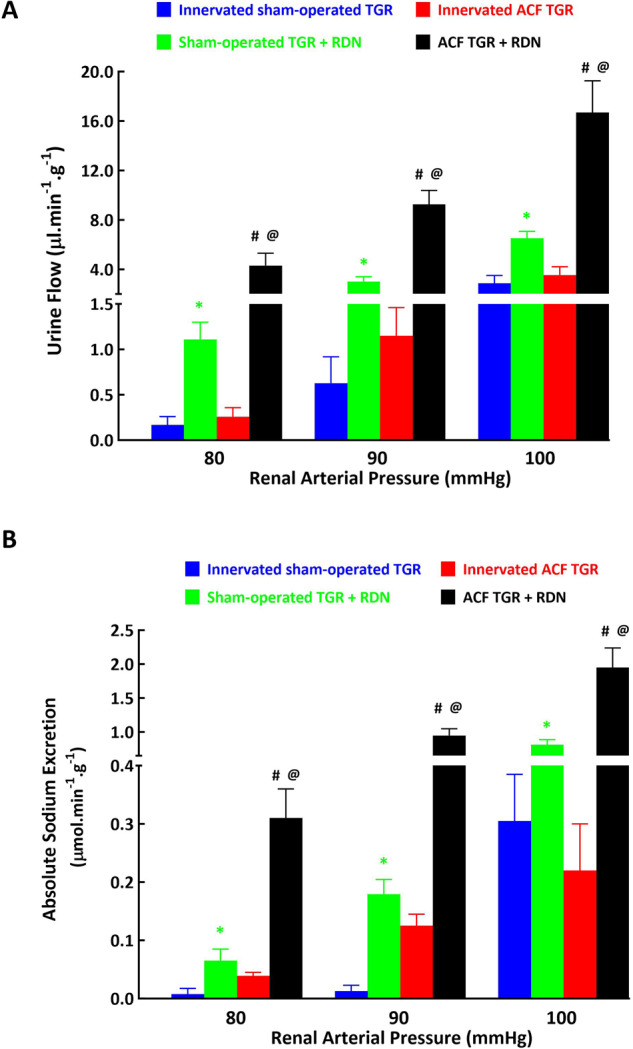
Urine flow (**A**) and absolute sodium excretion (**B**) at the renal arterial pressure (RAP) 100, 90 and 80 mmHg in innervated [i.e., without renal denervation (RDN)] sham-operated Ren-2 transgenic rats (TGR) (blue bars), in sham-operated TGR that underwent RDN (green bars), innervated TGR with aorto-caval fistula (ACF) (red bars) and ACF TGR with RDN (black bars). ^*^p < 0.05 compared sham-operated TGR + RDN with innervated sham-operated TGR. ^#^*p* < 0.05 ACF TGR + RDN compared with innervated ACF TGR. ^**@**^*p* < 0.05 ACF TGR + RDN compared with sham-operated TGR + RDN

Of special interest was that at low RAP (90 and 80 mmHg), in innervated ACF TGR the absolute values of urine flow were higher than observed in innervated sham-operated TGR (1.15 ± 0.31 vs. 0.63 ± 0.29 and 0.26 ± 0.09 vs. 0.16 ± 0.08 µl.min^–1^.g^–1^, *p* < 0.05 in both cases) (Fig. 11A), and the same was true for absolute sodium excretion (0.13 ± 0.02 vs. 0.013 ± 0.0.01 and 0.039 ± 0.006 vs. 0.074 ± 0.0 µmol.min^–1^.g^–1^, *p* < 0.05 in both cases) (Fig. 11B). It was the same with fractional sodium excretion (not shown in the figure).

As shown in Fig. 11A, B, the RDN-mediated attenuation of the decreases in urine flow and absolute sodium excretion in response to reductions in RAP to 100, 90 and 80 mmHg was markedly more prominent in ACF TGR as compared with the sham-operated TGR. This resulted in distinctly higher absolute values of these renal excretory parameters in denervated ACF TGR than observed in all the other experimental groups. Of particular interest is comparing absolute sodium excretion in ACF TGR + RDN group with sham-operated TGR + RDN group: it is seen that absolute sodium excretion in denervated ACF TGR is about 3-fold higher at 100 mmHg, about 6-fold higher at 90 mmHg and again about 5-fold higher at 80 mmHg than observed in sham-operated TGR + RDN group (Fig. 11B). The same is principally valid for urine flow (Fig. 11A) and also for fractional sodium excretion (data not shown in the figures).

## Discussion

The first important set of findings relates to the effects of RDN on autoregulatory capacity of renal hemodynamics and kidney pressure-natriuresis relationship. We found that efferent RDN did not improve the already well-established impairment of autoregulatory capacity of RBF and GFR in sham-operated TGR [40, 47], but attenuated to some extent the well-known suppressed pressure-natriuresis relationship in sham-operated TGR with established hypertension [40, 47, 65, 66]. These findings indicate that even if RSNA plays an important modulatory role in regulation of renal hemodynamics and renal tubular sodium reabsorption [20, 21], increased RSNA activity is not the cause of impaired autoregulatory capacity of renal hemodynamics and the abnormal pressure-natriuresis in TGR. This first conclusion agrees with the data from spontaneously hypertensive rats (SHR), the most common model of human primary hypertension. This shows that abnormal RSNA activity is not the primary cause of the abnormal pressure-natriuresis relationship in SHR with established hypertension [67].

Nevertheless, of special importance here is the observation that in ACF TGR, RDN enabled markedly better maintenance of autoregulation of RBF and GFR at lower levels of RAP (90 and 80 mmHg) than in innervated ACF TGR. This is of particular interest because the RAP of 80 mmHg is the lowest BP limit for maintaining relatively constant RBF and GFR in healthy, normotensive subjects [41, 68–70]. Thus, in ACF TGR, RDN protected the autoregulatory capacity of RBF and GFR at the critically important borderline values of RAP. Even more important, in ACF TGR, RDN markedly improved the pressure-natriuresis relationship as compared with all the other experimental groups; again, this was evident at lower levels of RAP (100, 90 and 80 mmHg), where urine flow and absolute and fractional sodium excretion were substantially higher than those in all the other groups. It is emphasized that all these beneficial actions of RDN on renal autoregulatory capacity and the pressure-natriuresis relationship in ACF TGR were seen even though RDN did not prevent the decrease in the basal RBF. Taken together, the findings are compatible with our initial hypothesis that even though in this model of cardiorenal syndrome RDN does not clearly prevent impairment of renal perfusion, it considerably improves the renal autoregulatory capacity and the pressure-natriuresis relationship. Consequently, the leftward shift of the pressure-natriuresis curve might facilitate sodium excretion under conditions of lower RAP in ACF TGR, which likely delays the onset of HF decompensation phase and probably is one of the mechanisms underlying RDN-induced attenuation of HF-dependent mortality in ACF TGR, as we reported recently [40].

Even though our present study does not allow us to determine the exact mechanism(s) responsible for the improvement of the pressure-natriuresis relationship in ACF TGR after RDN, it is important to consider some important facts. First, our previous studies demonstrated that intrarenal concentration of norepinephrine (NE) is elevated in innervated ACF TGR, however, the state of intrarenal adrenergic receptors (determined as kidney mRNA and protein expression and also by receptor ligand binding studies) were not altered [39, 71]. This indicated activation of RSNA in innervated ACF TGR because the rats exhibited increased NE concentration together with inappropriately maintained renal expression of adrenergic receptors. In addition, previous studies have shown that RDN caused more than 95% reduction in kidney NE concentration in ACF TGR [39, 71]. Since renal NE is an established neurochemical marker of RDN effectiveness [20, 21, 39, 54–56], the beneficial effects on the pressure-natriuresis relationship might depend on inhibition of inappropriately high RSNA and of its direct or indirect influence on the tubular water and sodium reabsorption.

Second, our previous findings showed that RDN did not alter the augmented intrarenal activity of RAS in ACF TGR [39, 71]. Noteworthy, even though ANG II is not a mediator of pressure-natriuresis mechanism, it is known to modulate it significantly, and alterations of intrarenal RAS activity critically influence the pressure-natriuresis curve [72, 73]. Therefore, improvement of the pressure-natriuresis relationship in ACF TGR in response to RDN cannot be ascribed to the changes of intrarenal RAS activity. Evidently, focused studies will be necessary to determine the mechanism(s) of actions of RDN on renal tubular water and sodium reabsorption in ACF TGR at this critical phase of HF.

Of considerable interest are the effects of changes in RAP on intrarenal hemodynamics. Previous clinical and experimental studies showed that in the early phase and compensated phase of HF, the RBF but not GFR was lowered, which resulted in increased FF, probably a sign of preferential vasoconstriction of the efferent arteriole mediated by activation of the RAS [6, 15–17, 74]. This helped maintain stable GFR, a prerequisite for satisfactory renal function. We showed that FF was increased in innervated ACF TGR, and at the physiological RAP level, RDN did not alter it. Since we saw that RDN did not modify intrarenal RAS activity, our present findings agree with the interpretation given above. We found that RAP reduction in innervated sham-operated TGR was followed by profound decreases in FF, particularly at lower levels of RAP (90 and 80 mmHg). Thus, the efferent arteriole responded to RAP reduction by more prominent vasodilatation as compared with the afferent arteriole, and because RDN attenuated these decreases in FF, RSNA might have some role in the response. In contrast, the changes in RAP did not alter FF in ACF TGR, either innervated or after RDN suggesting similar vasoactive actions on the afferent and efferent arterioles. This was unexpected because the efferent arterioles are usually thought not to participate in RBF autoregulation [41, 69, 70]. Admittedly, they may contribute to autoregulation of GFR at low RAP that is when intrarenal RAS is markedly activated, e.g., by low-sodium diet or also during ANG II administration [41, 69, 70]. Consequently, one would expect some role of the efferent arteriole, if any, in ACF TGR rather than in sham-operated TGR. It is reminded that FF alone is not an unequivocal index of the localization of resistance changes in the glomerulus: increases in FF can occur as a consequence of simultaneous increases in the afferent and efferent arterioles [72, 75]. In two different models of HF, low-output variant induced by myocardial infarction and high-output variant induced by ACF creation, FF was indeed increased. This suggested preferentially enhanced postglomerular resistance, but the subsequent renal micropuncture studies showed that both afferent and efferent arteriolar resistance (R_A_, R_E_) were comparably increased in both HF models [74]. However, other studies evaluating both whole kidney and single nephron function showed a striking correlation between FF level and R_A_ and R_E_ [76, 77]. Therefore, we admit that our present findings regarding changes of intrarenal hemodynamics in response to RAP changes based on FF alterations should be supplemented with renal micropuncture studies.

Another important finding in innervated ACF TGR was the dynamic pattern of changes in organ weights, autoregulatory capacity of the RBF, and the pressure-natriuresis relationship in the very early phase of HF (i.e., one week after ACF creation) compared with the phase of HF decompensation (i.e., three weeks after ACF creation).

Previous studies (including ours) have clearly demonstrated that organ weights (whole HW, LVW, RVW and lung weight) are reliable predictors of the onset of cardiac decompensation, at least in ACF-induced high-output HF model [31, 32, 38–40, 58, 78–81]. The organ weights data from the very early phase of HF in innervated ACF TGR were reported in our recent study [40]; for ethical reasons we decided to use those results for our current comparison analysis instead of repeating the experiments.

In both phases innervated ACF TGR showed increases in whole HW as compared with innervated sham-operated counterparts but the increase was more pronounced three weeks than one week after ACF creation (+51% versus +20%). The LV mass increase one week after ACF creation was only 2.5%, compared with 25% after three weeks. The increase in RV mass was markedly higher as compared with LV mass increases in both HF phases, but again it was more distinct three weeks than one week after ACF creation (+232% versus +78%). Our analyses at the tissue level showed that the heart remodeling response to chronic volume overload, i.e., development of eccentric LV hypertrophy (hallmark of the cardiac pathophysiology in this model of HF) was clearly present in innervated ACF TGR already one week after creation of ACF [50]. Our present data clearly show that the development of eccentric remodeling dynamically progressed three weeks after ACF creation, because when we compared the cardiomyocyte length to cardiomyocyte width ratio in innervated ACF TGR three weeks after ACF creation with the ratio obtained in innervated ACF TGR one week after ACF induction, we found that it was markedly higher (8.12 ± 0.68 vs. 6.541 ± 0.18, *p* < 0.05). The lung weight was already noticeably increased one week after ACF creation and, again, the increase in lung weight was more noticeable three weeks after ACF creation (+91% versus +49%). Our analysis shows that in the very early phase of HF the increase in whole HW is entirely due to an increase in RV mass. In the HF decompensation phase LV hypertrophy is also seen, but RV hypertrophy dominates.

Important lung congestion is present already in the very early phase of HF and in the phase of decompensation it is very pronounced. Our previous and present data are not conclusive regarding mechanism(s) underlying immediate and prominent lung congestion in innervated ACF TGR. In this context, it is important to remind that according to Linzbach, the transition to the decompensated HF is triggered by a marked ventricular dilatation once the myocardial compensatory response is achieved [82]. Therefore, we propose that the inability of the LV to develop hypertrophy comparable with that of RV might be one of the critical factors responsible for the development of overt HF in innervated ACF TGR. More comprehensive studies are needed to test this hypothesis.

In our recent study with innervated ACF TGR we found that in the very early phase of HF, RBF autoregulatory capacity was well-maintained up to the lowest level of RAP (80 mmHg), even though RBF was already lower than in their innervated sham-operated counterparts; also, the pressure-natriuresis relationship was improved [40]. We concluded that maintained RBF autoregulation capacity and the leftward shift of the pressure-natriuresis relationship is a compensatory mechanism, whereby the effects of the initial insult in this high-output HF variant is at least partially compensated and the progression to its overt form is retarded [40]. In our present study, we found that in the phase of HF decompensation the innervated ACF TGR displayed further decrease in RBF which was accompanied by a significant rise in RVR. Dissimilarly, in innervated ACF TGR in the very early phase of HF the RVR was not increased.

Noteworthily, autoregulatory capacity of RBF at lower levels of RAP (90 and 80 mmHg) was impaired in innervated ACF TGR three weeks after ACF creation, whereas one week after ACF creation it was well-maintained [40]. This is of crucial importance because RBF level determines the forces of glomerular ultrafiltration, of fluid reabsorption into the peritubular capillaries, and maintenance of the medullary concentration gradient [41, 68–70]. The pattern of the relationships is complex: it is known that GFR increases only moderately with increasing RBF, but decreases greatly with decreasing RBF [69, 70]. Nevertheless, three weeks after ACF creation innervated ACF TGR still exhibited altered pressure-natriuresis relationship, but at the lower RAP level (90 mmHg) this was not so prominent as in innervated ACF TGR one week after ACF creation [40]. Taken together, these findings show that three weeks after creation of ACF are required for the renal compensatory mechanism to come fully in action (as shown by the maximal leftward shift of the pressure-natriuresis relationship). On the other hand, the findings demonstrate that in this phase renal compensatory mechanisms are no more fully effective, as seen from the impairment of the autoregulatory capacity of RBF and GFR at lower RAP levels. Evidently, this is the stage when innervated ACF TGR enter the decompensation phase of HF.

### Limitations of the study

The first limitation is that the study focused exclusively on the pathophysiological role of efferent renal sympathetic nerves in the ACF-induced cardiorenal syndrome. However, the kidneys are also innervated by sensory (afferent) fibers that relay information to the brain to modulate sympathetic outflow [21]. Awareness is growing that increased activity of the renal afferent nerves (within the “kidney-brain neural circuit”) might also play important role in the pathophysiology of hypertension, kidney damage and HF [21, 83, 84]. It has to be recognized that our standard bilateral RDN technique results in ablation of afferent as well as efferent renal nerves and it is possible that some beneficial actions of RDN may have been mediated by interruption of the “kidney-brain neural circuit”. Therefore, it is evident that future studies are needed to evaluate the precise role of renal afferent nerves in the pathophysiology of cardiorenal syndrome.

The second limitation is that our study was performed in male animals only and it is now well-known that there are sex-linked differences in the pathophysiology and the course of HF and cardiorenal syndrome between males and females [3, 85]. Therefore, it is necessary to study the pathophysiological aspects of cardiorenal syndrome also in female animals and such studies are ongoing in our laboratory. This notion is also supported by recent recommendation that “sex” should no longer be an ignored experimental variable, and is an unavoidable parameter in preclinical research, prerequisite for successful translation of results into clinical practice [86].

The third limitation relates to some technical aspects of our experimental procedure. We cannot be 100% sure that in the process of placing ultrasonic transient-time flow probe on the renal artery some damage of renal nerves does not occur. If so, our “innervated rats” could exhibit some degree of “renal denervation”. Given our many years’ experience with the application of flow probes, we believe that such effect is marginal. Another technical limitation might be that the reductions in RAP were performed sequentially and not randomized, which also could under some circumstances influence the outcome of our renal functional studies.

Nonetheless, even considering all the aforementioned limitations, we are convinced that the results of the current study provide important information on the pathophysiology of cardiorenal syndrome.

## Conclusion

The present results show that RDN exhibited protective actions on the autoregulatory capacity of RBF and GFR and substantially improved the pressure-natriuresis relationship at the time when TGR (RAS-dependent hypertensive rats) with high-output HF-induced cardiorenal syndrome entered the HF decompensation phase. This suggests that at low RAP levels RDN-mediated improvement of the pressure-natriuresis relationship might facilitate water and sodium excretion and might be the mechanism responsible for retardation of HF decompensation phase.

### Supplementary information

Supplementary Fig. 1

Supplementary Fig. 2

Supplementary Figure Legend
