# Supplementary figures and images for: Renal sympathetic denervation improves pressure-natriuresis relationship in cardiorenal syndrome: insight from studies with Ren-2 transgenic hypertensive rats with volume overload induced using aorto-caval fistula
Source: Hypertens Res. 2024 Feb 2;47(4):998–1016. doi: 10.1038/s41440-024-01583-0 (PMC10994851; doi:10.1038/s41440-024-01583-0)

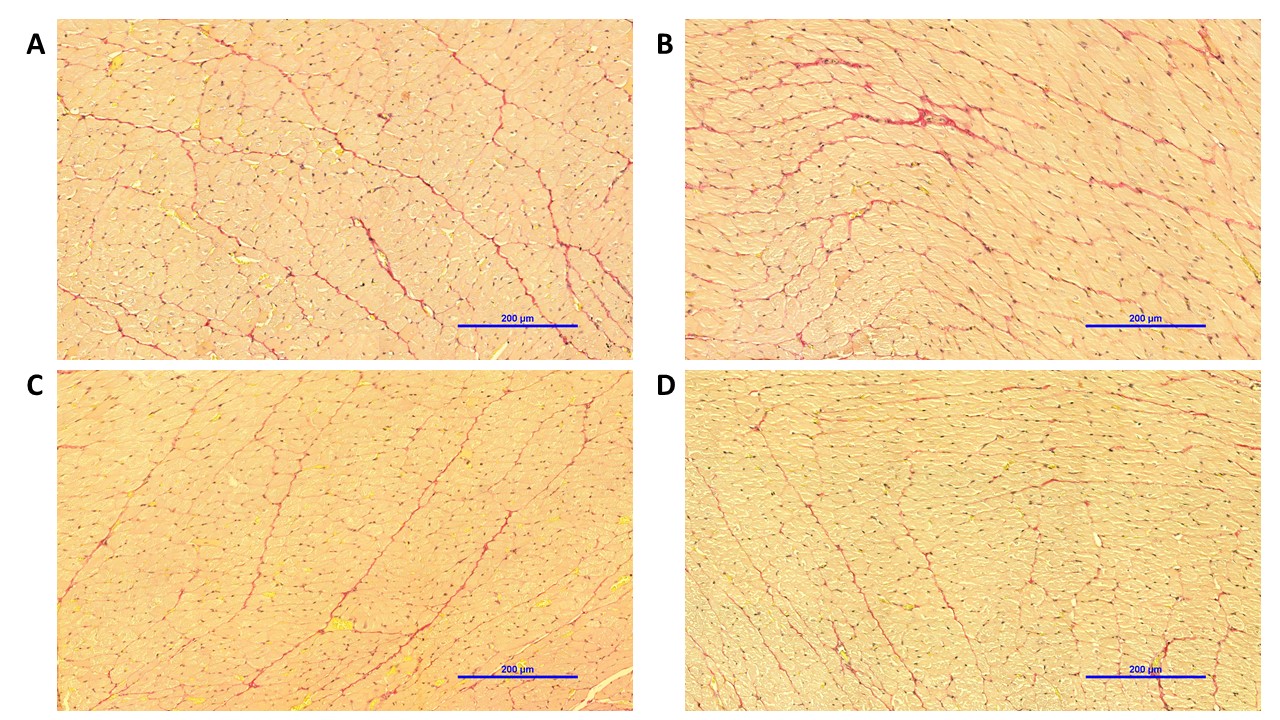

Supplement: Supplementary file 1 — Supplementary Fig. 1 [file 41440_2024_1583_MOESM1_ESM.jpg]

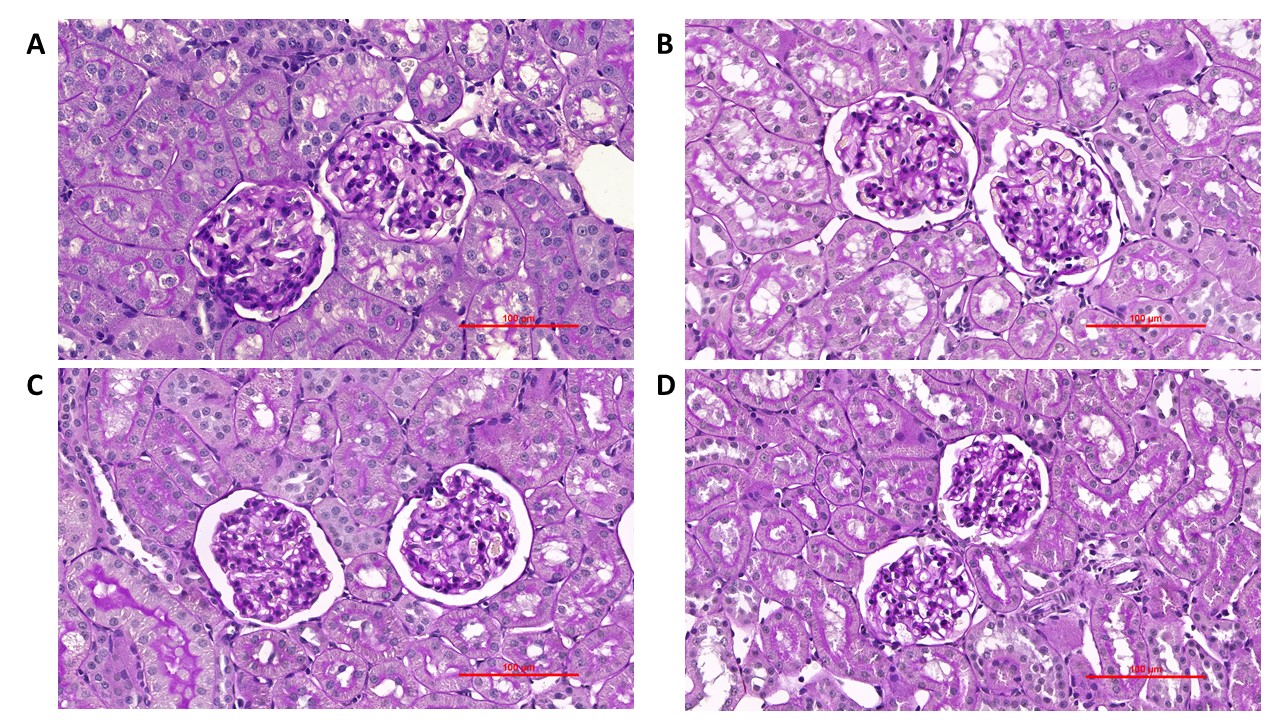

Supplement: Supplementary file 2 — Supplementary Fig. 2 [file 41440_2024_1583_MOESM2_ESM.jpg]
