# Supplementary material for: Renal sympathetic denervation improves pressure-natriuresis relationship in cardiorenal syndrome: insight from studies with Ren-2 transgenic hypertensive rats with volume overload induced using aorto-caval fistula
Source: Hypertens Res. 2024 Feb 2;47(4):998–1016. doi: 10.1038/s41440-024-01583-0 (PMC10994851; doi:10.1038/s41440-024-01583-0)
Supplement: Supplementary file 3 — Supplementary Figure Legend [file 41440_2024_1583_MOESM3_ESM.docx]

**Supplementary Figure 1.** Representative histological images of the left ventricle from the innervated sham-operated Ren-2 transgenic rats (TGR) [i.e., without renal denervation (RDN)] (A), in sham-operated TGR that underwent RDN (B), innervated TGR with aorto-caval fistula (C) and ACF TGR with RDN (D). The scale bar in the figure is 200 µm.

**Supplementary Figure 2.** Representative histological images of the renal cortex from the innervated sham-operated Ren-2 transgenic rats (TGR) [i.e., without renal denervation (RDN)] (A), in sham-operated TGR that underwent RDN (B), innervated TGR with aorto-caval fistula (C) and ACF TGR with RDN (D). The scale bar in the figure is 200 µm.
